# First Report of *Anguina pacificae* Parasitizing Turfgrass in Washington

**DOI:** 10.2478/jofnem-2025-0053

**Published:** 2025-11-27

**Authors:** Emily Braithwaite, Chas J. Schmid, Alec R. Kowalewski, Katherine Fleming, Amy B. Peetz, Inga A. Zasada, Hannah M. Rivedal

**Affiliations:** Department of Botany & Plant Pathology, Oregon State University, Corvallis, OR 97331; Department of Horticulture, Oregon State University, Corvallis, OR 97331; Horticultural Crops Disease and Pest Management Research Unit, United States Department of Agriculture – Agricultural Research Service, Corvallis, OR 97331; Forage Seed and Cereal Research Unit, United States Department of Agriculture – Agricultural Research Service, Corvallis, OR 97331

**Keywords:** *Anguina pacificae*, detection, *Poa annua*, turfgrass

## Abstract

The Pacific shoot-gall nematode (*Anguina pacificae*) is an economically important pest of annual bluegrass (*Poa annua L.*) putting greens in the coastal areas of northern and central California. In December 2024, diagnostic samples submitted to the Oregon State University Turfgrass Diagnostic Clinic (Corvallis, OR) from a golf course tee box in Clark County, Washington contained *A. pacificae*. Visual symptoms of chlorotic patches and dieback of the turf surface were observed, as well as swellings in the crowns that contained second-stage juveniles (J2). Morphological features as well as morphometric measurements of J2 were consistent with *A. pacificae*. Sequencing of the internal transcribed spacer (ITS) and the mitochondrially encoded *cytochrome c oxidase I (COX1)* gene regions confirmed the species identity. To our knowledge, this is the first report of *A. pacificae* parasitizing turfgrass in Washington.

The Pacific shoot-gall nematode (*Anguina pacificae* Cid del Prado Vera and Maggenti, 1984) is a devastating pest of golf course putting greens, specifically annual bluegrass (*Poa annua* L.; ABG). It has a narrow geographic range, currently only reported in North America in Northern California coastal climates (McClure et al., 2008) and on an ABG putting green in Ireland (Fleming et al., 2015). *A. pacificae* is a foliar-feeding endoparasitic nematode of cool-season turfgrass. The infective second-stage juvenile (J2) moves via films of water on the surface of the plant, enters the shoots, and causes a swelling or gall to develop at the crown of the plant as it feeds (Giat et al., 2008). The nematode continues its life cycle within the gall, eventually developing into an adult, and reproducing via amphimixes. A female can lay up to 1,200 eggs in each gall, many of which hatch within the gall into a J2. After several weeks, the gall begins to rot, and the J2 and remaining unhatched eggs are released and begin searching for nearby shoots to continue infection and development. Damage symptoms associated with *A. pacificae* are very different to many other turfgrass nematodes, because they affect the crowns and shoots of the plant, rather than the roots. Early symptoms include small, chlorotic patches anywhere from 25 mm to 75 mm in diameter that begin to coalesce as the damage spreads (McClure et al., 2008), often in patterns reflective of drainage lines, low-lying areas, or where water moves readily on the green. Symptoms of *A. pacificae* can be mistaken for common fungal pathogens in cool-season grasses because of their patch-like appearance, and severe infestations can lead to turf death, causing pitting or voids in the canopy that affect surface quality and playability, resulting in significant costs for management and recovery (Fleming et al., 2015).

In December 2024, diagnostic samples from an ABG golf course tee box in Clark County, Washington were sent to the Oregon State University Turfgrass Diagnostic Lab (Corvallis, OR) for disease analysis. The turfgrass exhibited symptoms of decline for several months, with circular patches of chlorotic tissue followed by thinning of the canopy, and eventual dieback (Fig. 1A). Initially, it was suspected that fungal pathogens such as summer patch (*Magnaporthiopsis poae*) were responsible for visual symptoms, but incubation and microscopic analysis of the sample showed no evidence of fungi. Additionally, standard fungicide applications for turfgrass root pathogens were not successful in improving turfgrass health. Furthermore, turf cores that included soil, root, thatch, and ABG tillers were collected from the tee box to assess plant-parasitic nematode population densities. Close examination of tillers revealed galling and swellings at the base of the stems (Fig. 1B), consistent with *A. pacificae* infection (Cid del Prado Vera and Maggenti, 1984). Baermann funnel extraction indicated approximately 30 J2/100 cm^3^ of soil and tiller material.

**Figure 1:**
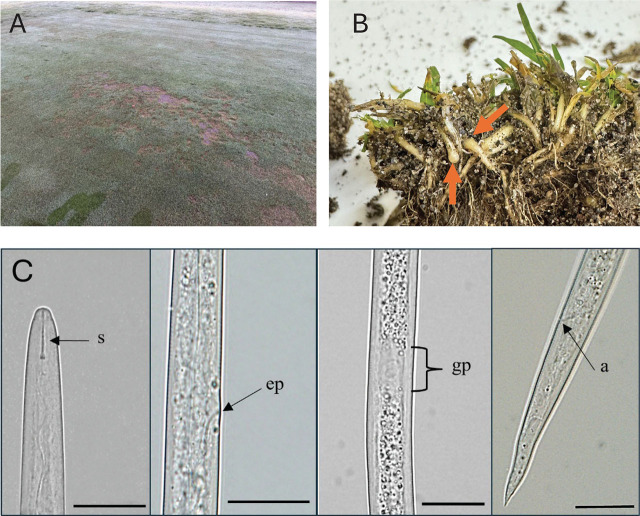
(A) Visual damage symptoms from the Pacific shoot-gall nematode, *A. pacificae*, on a golf course tee box in Clark County, Washington. (B) Basal galling and swelling of annual bluegrass (*P. annua* L.) tillers from the tee box. (C) *A. pacificae* second-stage juvenile. Scales: 200 μm. S, stylet; EP, excretory pore; GP, genital primordia; A, anus.

Stem galls were opened and J2 were liberated and hand-picked for morphological characterization. Nematodes were mounted, photographed, and measured using an Olympus BX51 microscope (Melville, NY) with an Olympus DP72 camera (Center Valley, PA). The following measurements (mean μm ± standard deviation) were determined: body length was 649.6 μm ± 51.9, body width 15.5 μm ± 1.2, stylet was weak with a length 10.4 μm ± 0.7, and knobs were rounded (Fig. 1C). The characteristic clearing in the mid-body for the genital primordium was observed with a length of 15.9 μm ± 4.1 and width of 9.0 μm ± 1.6. Measurements of the *Anguina* sp. population from Washington collected from ABG shoot-galls were within the range reported for *A. pacificae* (Cid del Prado Vera and Maggenti, 1984; Fleming et al., 2015).

To confirm the identification of *A. pacificae*, DNA was extracted from single J2 (*n* = 10). Individual nematodes were picked and lysed using methods described by Peetz and Zasada (2016). Primers rDNA1/rDNA2 (5′-TTGATTACGTCCCTGCCCTTT-3′/5′-TTTCACTCGCCGTTACTAAGG-3′; Vrain et al., 1992), and COIF/COIR (5′-GATTTTTTGGKCATCCWGARG-3′/5′-CWACATAATAAGTATCATG-3′; Lazarova et al., 2006) were used to amplify the internal transcribed spacer (ITS) for five individuals (these were the same individuals used for the above described imaging), and mitochondrially encoded cytochrome c oxidase I (COX1) for five other individuals. Cycling conditions for ITS included an initial denaturation step of 94°C for 3 min, followed by 40 cycles of 94°C for 1 min, an annealing temperature of 55°C for 1 min, 72°C for 1 min, and a final extension at 72°C for 10 min. For the COX1 region, cycling conditions included an initial denaturation step of 95°C for 3 min, followed by 35 cycles of 95°C for 30 sec, an annealing temperature of 53°C for 30 sec, 72°C for 45 sec, and a final extension at 72°C for 7 min. A 10 μl aliquot of each PCR reaction was analyzed by electrophoresis on 1.5% agarose/Tris-acetate-EDTA (1X TAE) gels stained with GelRed^®^ nucleic acid gel stain (Biotium, San Francisco, CA), and visualized by UV illumination. Amplification of these gene regions resulted in a single band of ~1,000 bp for ITS and ~450 bp for COX1. PCR amplicons were enzymatically cleaned using ExoSAP-IT PCR Product Cleanup Reagent (Applied Biosystems, Carlsbad, CA) according to the manufacturer’s protocol and sequenced bidirectionally using standard methods by Eurofins Genomics LLC (Louisville, KY). Resultant sequences were trimmed and pairwise aligned to generate a consensus sequence in Geneious software version 2022.2.2 (Biomatters, Auckland, NZ). Identities of each sequence were determined using the National Center for Biotechnology Information (NCBI) Basic Local Alignment Search Tool (BLAST) for nucleotide sequences and submitted to NCBI GenBank (ITS accession numbers PV537251-PV537255; COX1 accession numbers PV537257-PV537261). The BLASTn analysis of ITS sequences matched with *A. pacificae* accession KP715099.1 with 100% identity and an *E*-value of 0. The BLASTn analysis of COX1 sequences resulted in no matches to *A. pacificae* due to the lack of reference sequences in the NCBI database. There were, however, matches to other Anguinidae reference sequences with identities of 80% (*Subanguina danthoniae*; MW086880.1) and 84% (*Anguina agrostis*; MG321205.1), suggesting that while the query sequences likely belong to a member of the family Anguinidae, the absence of *A. pacificae* in the reference database limits precise species-level identification. Because of the lack of reference sequences for COX1, a phylogenetic tree was constructed only from the amplified region of ITS, as well as sequences of Anguinid reference isolates obtained from NCBI. Multiple sequence alignments (MSA) of the ITS sequences were constructed using MAFFT v. 7.394 with the G-INS-I algorithm (Katoh and Standley, 2013), and the resultant MSA was manually corrected. A maximum likelihood tree reconstruction was performed using randomized axelerated maximum likelihood (RAxML; Stamatakis, 2014) with 100 bootstraps. Each phylogenetic reconstruction was performed under the GTR-GAMMA I model, using “rapid bootstrapping and searching for the best scoring maximum likelihood tree” setting in Geneious. The resulting phylogenetic tree placed the *A. pacificae* sequences from ABG in Washington with other sequences of *A. pacificae* with 100% bootstrap support (Fig. 2), supporting the results described above.

**Figure 2:**
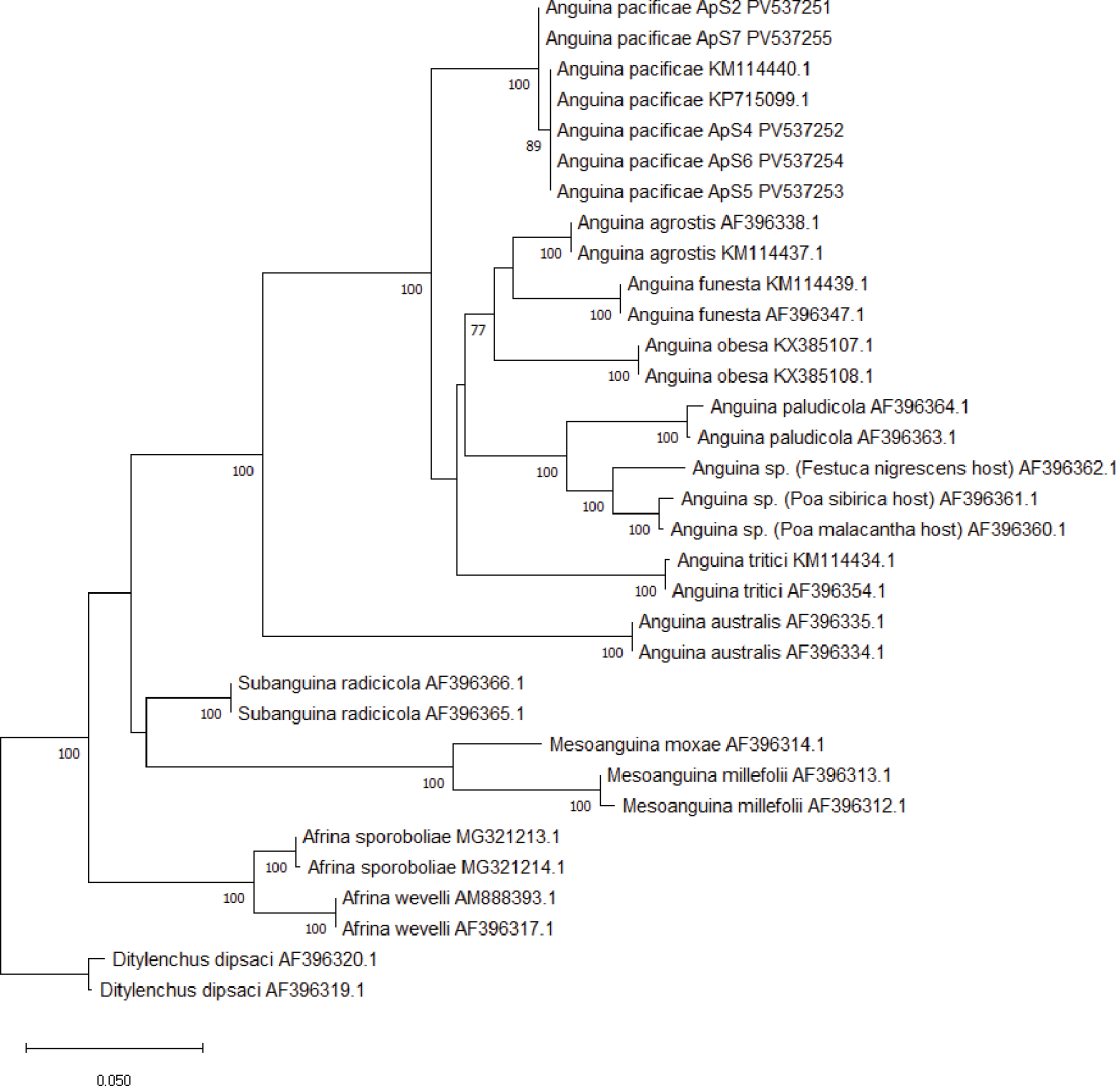
Phylogenetic tree, derived by RAxML analysis of the ITS gene region for *A. pacificae*. Alignments and trees were constructed in Geneious using MAFFT alignment tool. The tree was run with 100 bootstraps, only bootstrap support >70% are shown to increase readability. ITS, internal transcribed spacer; RAxML, randomized axelerated maximum likelihood.

This is the first report of *A. pacificae* on ABG in Washington. This nematode has previously been found on multiple ABG putting greens in California (Cid del Prado Vera and Maggenti, 1984; Giat et al., 2008; McClure et al., 2008) and a single golf course in Ireland (Fleming et al., 2015), but this is the first report on higher mown turfgrass of a sand-based tee box. This documentation of *A. pacificae* in Washington indicates movement north from the initial California detections. Given that ABG is the predominant putting green surface throughout the Pacific Northwest (including Oregon and Washington), further surveys are warranted to determine whether this is an isolated case or if *A. pacificae* is more widely distributed in the region than currently recognized.
